# Socio-demographic characteristics associated with SF-6D v2 utility scores in patients undergoing dialysis in China: contributions of the quantile regression

**DOI:** 10.1186/s12955-025-02401-y

**Published:** 2025-07-28

**Authors:** Ye Zhang, Li Yang, Zeyuan Chen

**Affiliations:** 1 Population Development Studies Center, Renmin University of China, Beijing, 100872 People’s Republic of China; 2 School of Population and Health, Renmin University of China, Beijing, 100872 People’s Republic of China; 3 School of Public Health, Peking University, Beijing, 100191 People’s Republic of China; 4 Department of Informatics and Media, Uppsala University, Uppsala, SE-751 05 Sweden

**Keywords:** Dialysis, Health-related quality of life, Quantile regression, SF-6Dv2, Utility scores

## Abstract

**Background:**

Generic preference-based instruments, such as the Short Form 6-Dimensions (SF-6D) and EuroQol 5-Dimensions (EQ-5D), can generate utility scores that facilitate the estimation of health-related quality of life (HRQoL) which is commonly used in cost-utility analysis. This study investigated the associations between utility scores and potential socio-demographic factors in Chinese patients with dialysis using quantile regression.

**Methods:**

Patients were recruited in a multicenter survey conducted between November 2023 and January 2024 for dialysis patients in China. Patient responses to the SF-6D version 2 (SF-6Dv2) instruments were used to calculate utility scores. The relationships between utility scores and potential socio-demographic factors were examined using both ordinary least squares (OLS) and quantile regression models. The Wald test was employed to test the differences in coefficients across quantiles in quantile regression. Model performance was assessed using 5-fold cross-validation.

**Results:**

A total of 378 patients were included. Age, education level, having a loan due to illness, currently working, monthly income > 8000 RMB and number of comorbidities were associated with utility scores. The quantile regression coefficients and Wald test suggested that the size of the associations between the utility scores and factors varied along with the utility score distribution. Quantile regression yielded more accurate fitted and predicted values compared to OLS regression.

**Conclusion:**

Quantile regression offers a valuable complement in analyzing factors associated with utility scores among Chinese dialysis patients. For policymakers, differentiated nonclinical strategies may be needed to improve HRQoL across varying health states within this population.

**Supplementary Information:**

The online version contains supplementary material available at 10.1186/s12955-025-02401-y.

## Introduction

Health related quality of life (HRQoL) has been widely used to assess an individual’s health status. It can be measured by both generic preference-based measures and disease-specific measures. The generic preference-based measures, such as EQ-5D and SF-6D, are able to provide both different dimensions of a patient’s health-related state (e.g., social functioning and anxiety) and a single, comparable utility score for a patient’s HRQoL [1]. Because it is highly interpretive and comparable, the utility score is commonly used in clinical research, cost measurement, and decision making [2]. 

Chronic kidney disease (CKD) is a progressive condition characterized by high incidence, high disability rates, and long treatment period [3]. Thus, patients undergoing CKD often experience a decline in HRQoL. In China, CKD affects approximately 8.2% of the adult population in 2018 [4], amounting to over 82 million individuals, with the expected number of patients receiving dialysis treatment exceeding 800,000 in 2025 [5]. This poses a significant public health burden and highlights the importance of assessing HRQoL in this population. A meta-analysis on the EQ-5D-5L reported that CKD patients, especially when undergoing dialysis, have significantly impaired HRQoL, particularly in the self-care dimension [6]. Another study conducted in China reported the mean EQ-5D-5L and SF-6D scores among patients with end-stage renal disease (ESRD) was 0.68 and 0.70, respectively in 2014 [7]. Several studies have explored the association between HRQoL and demographic and socioeconomic characteristics in dialysis patients [8–12]. However, findings across studies have not always been consistent. For example, some studies have found a positive correlation between education level and HRQoL [9], while others have noted that subjects with higher levels of education instead have lower HRQoL [13]. For the variable of work status, some studies have found that employed dialysis patients report better HRQoL [8, 11], while others have concluded that there is no association between having a job or not and HRQoL [10]. Furthermore, the majority of research employs disease-specific measures to assess HRQoL which do not allow calculating utility scores.

Regarding the statistical methods to study these associations, ordinary least squares (OLS) are the most commonly used [14]. However, the distribution of utility scores among patients may exhibit skewness and lead to poor model fit and reduce the predictive accuracy. Some studies have reported that OLS fails to adequately capture the extremes of the utility score distribution (i.e., those in very good or very poor health) [15]. Unlike OLS, which focuses on the mean, quantile regression allows exploring the variations in associations along with the distribution of the utility score, especially at the extremes of the distribution [16]. This is particularly important because OLS estimates can be biased or less efficient at the tails when data deviate from normality, whereas quantile regression directly models different points of the outcome distribution, capturing heterogeneity more effectively. While studies have explored the advantages of quantile regression over OLS in other disease areas, to the best of our knowledge, no research has yet applied quantile regression on the HRQoL related studies in kidney disease patients.

Compared with other instruments, SF-6Dv2 improves the measurement precision of certain health dimensions, including mental health and social functioning, and provides higher discriminative ability with less ceiling effect. These advantages make SF-6Dv2 more suitable for capturing HRQoL variations in chronic disease populations. Given these improvements, SF-6Dv2 was selected in this study to more accurately reflect the utility profiles of dialysis patients in China.

This study aimed to investigate the relationships between utility scores and potential factors among dialysis patients in China using both OLS and quantile regression models. We also compare the performance of the two models.

## Methods

### Data source and study population

The data were collected from a multicenter survey conducted between November 2023 and January 2024 for dialysis patients in China. The questionnaire was distributed through the largest online survey platform in China, Wen Juan Xing (Changsha Ranxing Information Technology Co., Ltd., Hunan, China). Participants mainly were outpatients admitted to eight hospitals in four big cities (Beijing, Xian, Chengdu, and Hangzhou) in China and the participating hospitals were the main nephrology centers of each city. To prevent selection bias due to electronic incompetence, face-to-face interviews were used to help complete the survey for those patients who were unable to use electronic devices. To reach the largest possible number of participants, the survey was also distributed in dialysis patient WeChat groups which have been certified by the relevant nephrologist from the hospital we included. With this, we aimed to reach a wider and different sample of patients who have experienced dialysis from hospitals at different levels in different regions of China. The inclusion criteria of the participants who (1) were undergoing kidney dialysis treatment, (2) were going through dialysis therapies for at least three months, (3) were able to communicate normally and independently, (4) agreeing to participate in the study, and (5) had given informed consent. The exclusion criteria were patients with mental illness or cognitive impairment. The study was approved by the Ethics Committee for Research at Renmin University of China.

### SF-6Dv2 utility score

HRQoL was assessed using the Chinese version of the Short Form 6-Dimensions (SF-6Dv2). The The SF-6Dv2 improves upon its predecessor (SF-6Dv1) by refining response levels. SF-6Dv2 contains 6 domains (physical function, role limitations, social function, mental health, bodily pain, and vitality) with 5 or 6 response levels for each domain [17, 18]. Therefore, the SF-6Dv2 has a total of 18,750 possible responses. Its validity and reliability in patients with non-communicable diseases have been demonstrated in prior studies. Value sets for the SF-6Dv2 have been developed in multiple countries and regions, including China, Japan, Iran, the UK, Australia, and Quebec. The Chinese value set for SF-6Dv2 has been developed using the composite TTO approach in 2020, where SF-6Dv2 utility scores range from − 0.277 (worst health state) to 1.0 (best health state) [17]. 

### Potential associated factors

The demographic characteristics included sex, age, and marital status (current spouse or not). The socioeconomic variables included education level (primary and below; middle school; high school; undergraduate and above), currently working, monthly income above 8000 RMB, having a loan due to illness, and receiving government aid. In addition, we also included clinical and health behavior-related variables, such as the number of comorbidities, being a habitual smoker, and being a habitual drinker.

### Statistical analysis

Descriptive analyses were conducted to summarize potential associated factors (frequency and percentage) and SF-6Dv2 responses (mean, standard deviation, and median for subgroups defined by these factors). Shapiro-Wilk normality tests were employed to determine the distribution of SF-6Dv2 scores.

For the univariate analyses, Mann-Whitney U test (for factors with two subgroups), and Kruskal-Wallis test (for factors with three or more subgroups) and spearman rank correlation test (for age) were used to test the differences of the mean SF-6Dv2 scores across subgroups of associated factors.

Nonparametric tests were used because the data failed the normality test. OLS regression and quantile regression models were then fitted to the sample. The dependent variable was the SF-6Dv2 utility score. Independent variables were associated factors that have p-values less than 0.1 in the univariate analyses. We performed a quantile regression carrying out five models based on the median (Q50), the first (Q25), and third (Q75) quartiles and the first (Q10) and ninth (Q90) deciles. These five quintiles have been widely used in previous studies and are considered to be representative of the entire distribution [1, 19, 20]. We obtained coefficient estimates (CEs) and their 95% confidence intervals (CIs) for each quantile model, and a trend chart was presented for each variable. A variable was considered significant when it reported a p value below 0.1. For each variable, we investigated differences between each pair of quantile CEs using the Wald test [21]. The presence of differences suggests that the magnitude of the relationship between SF-6Dv2 scores and potentially relevant factors varies by quartiles.

We compared the performance of OLS regression model and quantile regression models by 5-fold cross-test on the dataset. The mean absolute error (MAE) and the root mean squared error (RMSE) were calculated to show the predictive accuracy of the models, with lower MAE and RMSE representing better predictive accuracy. For quantile regression, we calculated MAE and RMSE for each quantile model. However, in order to gain a global measure to generally assess the model performance and to compare quantile regression with OLS, we designed a “best estimate” model whose predicted value is the closest to the observed value among the predicted values of the five quantile models [1]. Model fitting on the full sample set (*n* = 378) was employed, and scatter plots of fitted and observed SF-6Dv2 scores were plotted to visually assess the goodness-of-fit of OLS and quantile regression. On the scatterplots of quantile regression (“best estimate”) fitting results we labeled which quantile model each best-fit value came from.

All the analyses were performed using R software version 4.4.1 (R Core Team, Vienna, Austria).

## Results

### Descriptive Statistics

Overall, 378 patients were included in this study. Age ranged from 17 to 87 years old and averaged 49.05 years old [standard deviation (SD), 13.34 years old]. The other characteristics of the study population are described in Table 1.

**Table 1:** SF-6Dv2 utility score by potentially associated factors (*N* = 378)

Potentially associated factors	*N*	%	SF-6D v2 utility Score			
			Mean	SD	Median	*P* value
**Sex**						0.729
Male	187	49.5%	0.579	0.252	0.638	
Female	191	50.5%	0.557	0.273	0.647	
**Currently with a spouse**						0.576
Yes	266	70.4%	0.561	0.265	0.643	
No	112	29.6%	0.585	0.258	0.650	
**Currently working**						**< 0.001**
Yes	66	17.5%	0.691	0.226	0.716	
No	312	82.5%	0.542	0.263	0.617	
**Education level**						**0.007**
Primary and below	32	8.4%	0.454	0.332	0.542	
Middle school	96	25.4%	0.554	0.253	0.628	
High school	110	29.1%	0.555	0.265	0.613	
Undergraduate and above	140	37.1%	0.614	0.242	0.676	
**Monthly income above 8000 RMB**						**0.083**
Yes	54	14.3%	0.622	0.243	0.653	
No	324	85.7%	0.559	0.265	0.635	
**Having a loan due to illness**						**0.076**
Yes	125	33.1%	0.533	0.287	0.608	
No	253	66.9%	0.585	0.249	0.650	
**Receiving government aid**						0.991
Yes	119	31.5%	0.578	0.258	0.630	
No	259	68.5%	0.564	0.265	0.650	
**Habitual smoker**						0.357
Yes	53	14.0%	0.597	0.251	0.656	
No	325	86.0%	0.563	0.265	0.643	
**Habitual drinker**						0.134
Yes	33	8.7%	0.652	0.220	0.667	
No	345	91.3%	0.560	0.265	0.642	
**Number of Comorbidities**						**< 0.001**
0	37	9.8%	0.650	0.275	0.708	
1	136	36.0%	0.605	0.239	0.653	
2	127	33.6%	0.570	0.257	0.650	
3	56	14.8%	0.506	0.243	0.564	
4 and above	22	5.8%	0.348	0.335	0.307	

Note: Bold indicates p values < 0.1; *SD* Standard deviation

The mean and median SF-6Dv2 scores were 0.538 and 0.608 (SD, 0.271), respectively. The SF-6Dv2 score ranged from − 0.267 to 1. Only 3 patients reported a utility of 1. Table A1 [see Additional File 1] presents the distribution of SF-6Dv2 levels across dimensions. In each dimension, fewer than 40% of participants reported the highest level (Level 1), further supporting the absence of a notable ceiling effect. According to the result of the Shapiro-Wilk normality test, the normality assumption of the SF-6Dv2 score distribution was rejected (*p* < 0.001). Spearman rank correlation coefficient was calculated to determine the potential correlation between age and SF-6Dv2 score (*r* = -0.2545, *p* < 0.001).

### Development of regression models

According to the results of the univariate analyses (Table 1), we included six variables in the multivariate regression models: age, education level, monthly income above 8000 RMB or not, having a loan due to illness or not, currently working or not, and number of comorbidities.

**Fig. 1:**
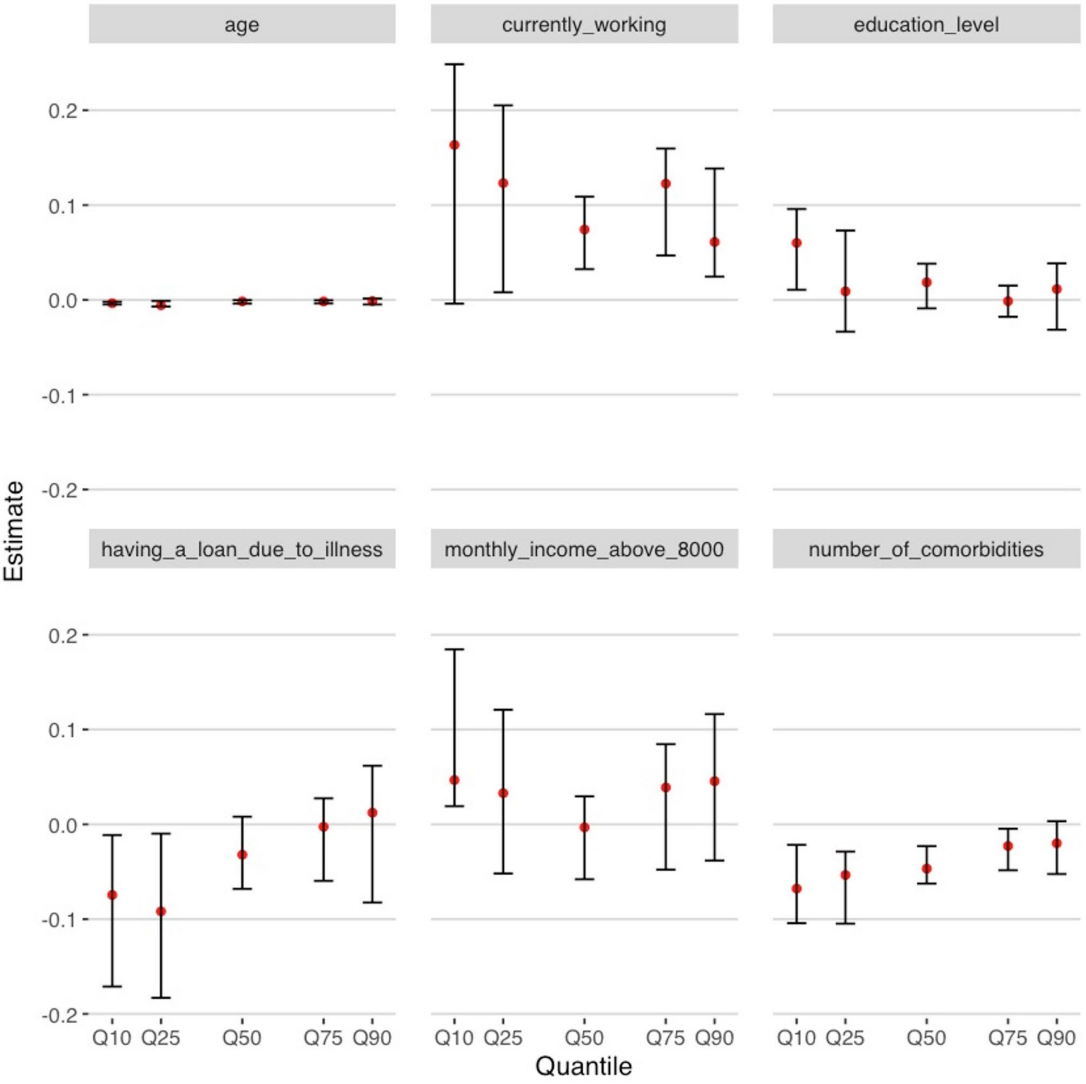
Results of the quantile regression (Q10, Q25, Q50, Q75 and Q90). **Note**. Estimates; − 95% confidence interval; Q10 first decile; Q25 first quartile; Q50 median; Q75 third quantile; Q90 ninth decile

The results of the OLS and quantile regressions are displayed in Table 2; Fig. 1. The CE and their 95% CI for all the variables included in the quantile regression can be seen in Table A2 [see Additional File 1]. The results of OLS model showed that individuals currently working, those have a loan due to illness, those with high age, and those with a high number of comorbidities had lower SF-6Dv2 score (*p* < 0.1). The results of quantile regressions showed that the variables education level, number of comorbidities, age and currently working might significantly influence SF-6Dv2 score (*p* < 0.1), and for different quantiles, the effect size (described by the magnitude of the CE in the quantile regression) of these variables was different. It can be seen from Fig. 1 that the variables education level and having a loan due to illness influence SF-6Dv2 larger on the left side of the distribution (Q10 and Q25).The number of comorbidities is more associated with lower SF-6Dv2 scores for the models with the inference based on Q10, Q25 and Q50. The absolute values of the CEs for the variables currently working and monthly income above 8000 RMB were relatively higher on the two sides of the SF-6Dv2 score distribution. The age variable was significant in all QR models, while its coefficient was slightly higher on the left side of SF-6Dv2 score’s distribution.

**Table 2:** Coefficient estimates (CE) for the OLS regression model

Potentially associated factors	CE	Standard errer	*P* value
Constant	0.7573	0.0748	**< 0.001**
Education level	0.0126	0.0141	0.371
Monthly income above 8000 RMB (Yes = 1)	0.0356	0.0378	0.346
Age	-0.0032	0.0010	**0.002**
Currently working (Yes = 1)	0.0948	0.0360	**0.008**
Comorbidities number	-0.0422	0.0131	**0.001**
Having a loan due to illness (Yes = 1)	-0.0485	0.0283	**0.087**

Note: Bold indicates P value < 0.1

The p-value of the Wald test for the five QR models is 0.015, meaning that it can be assumed that there is a difference between the CEs across quantiles. The results of Wald test by variables are shown in Table A3 [see Additional File 1]. The CE values for all variables showed significant differences between one or the other two models. For example, the CE for Education level was significantly different between the QR model based on Q10 inference and the QR models based on Q50 and Q75 inference; the CE for Having a loan due to illness was significantly different between the QR model based on Q25 inference and the QR models based on Q75 and Q90 inference. These differences illustrated the extent to which SF-6Dv2 scores were affected by the potential associated factors varied depending on their position in the distribution of HRQoL. Overall, the difference in regression coefficients between the QR model with inference based on Q25and those based on Q75 and Q90 was the most pronounced. These performances provided evidence of the heterogeneity of the independent variable’s impact on patients’ quality of life when they were in different positions of the SF-6Dv2 score distribution.

### Comparison of the models’ performances

The R^2^ for the OLS regression was 0.1257 while the pseudo R^2^, which is defined as the square of the Pearson correlation coefficient between predicted and observed values, for the 5 QR models ranged between 0.1053 and 0.1233. These results suggest that a large portion of the variance remains unexplained. The MAE and the RMSE were lower for the quantile regression when the “best estimate” of the five models is considered (Table 3).

**Table 3:** Results of 5-fold cross-validation of OLS regression and of the quantile regression (*N* = 226)

	OLS regression	Quantile regression					
		Q10	Q25	Q50	Q75	Q90	Best estimate
**MAE**	0.1929	0.4217	0.2880	0.2544	0.2989	0.3613	**0.0600**
**RMSE**	0.2505	0.3707	0.2423	0.1847	0.2140	0.2798	**0.0981**

Note: Bold indicates the optimal results; *MAE* Mean absolute prediction error; *RMSE* Root mean squared prediction error

**Fig. 2:**
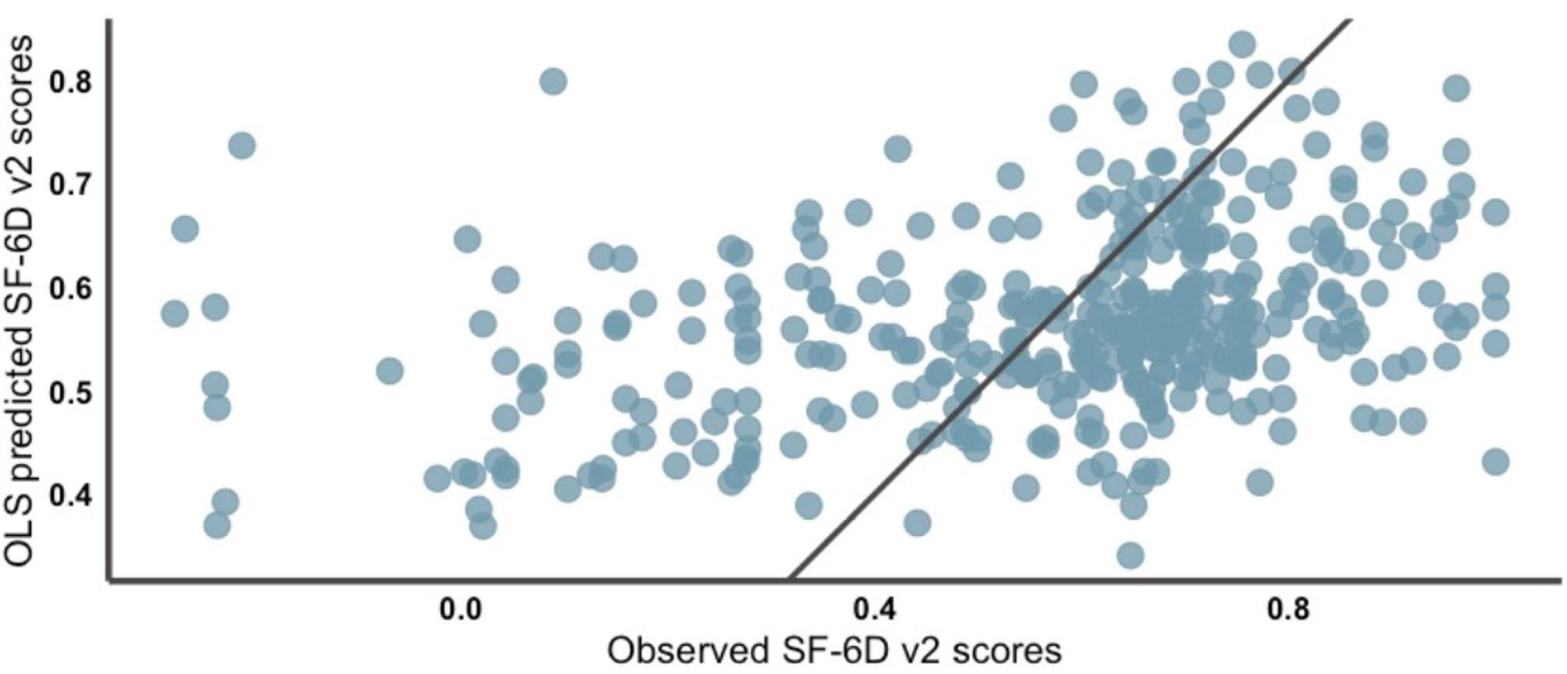
Scatter plot of prediction vs. observation resulting from the OLS regression

**Fig. 3:**
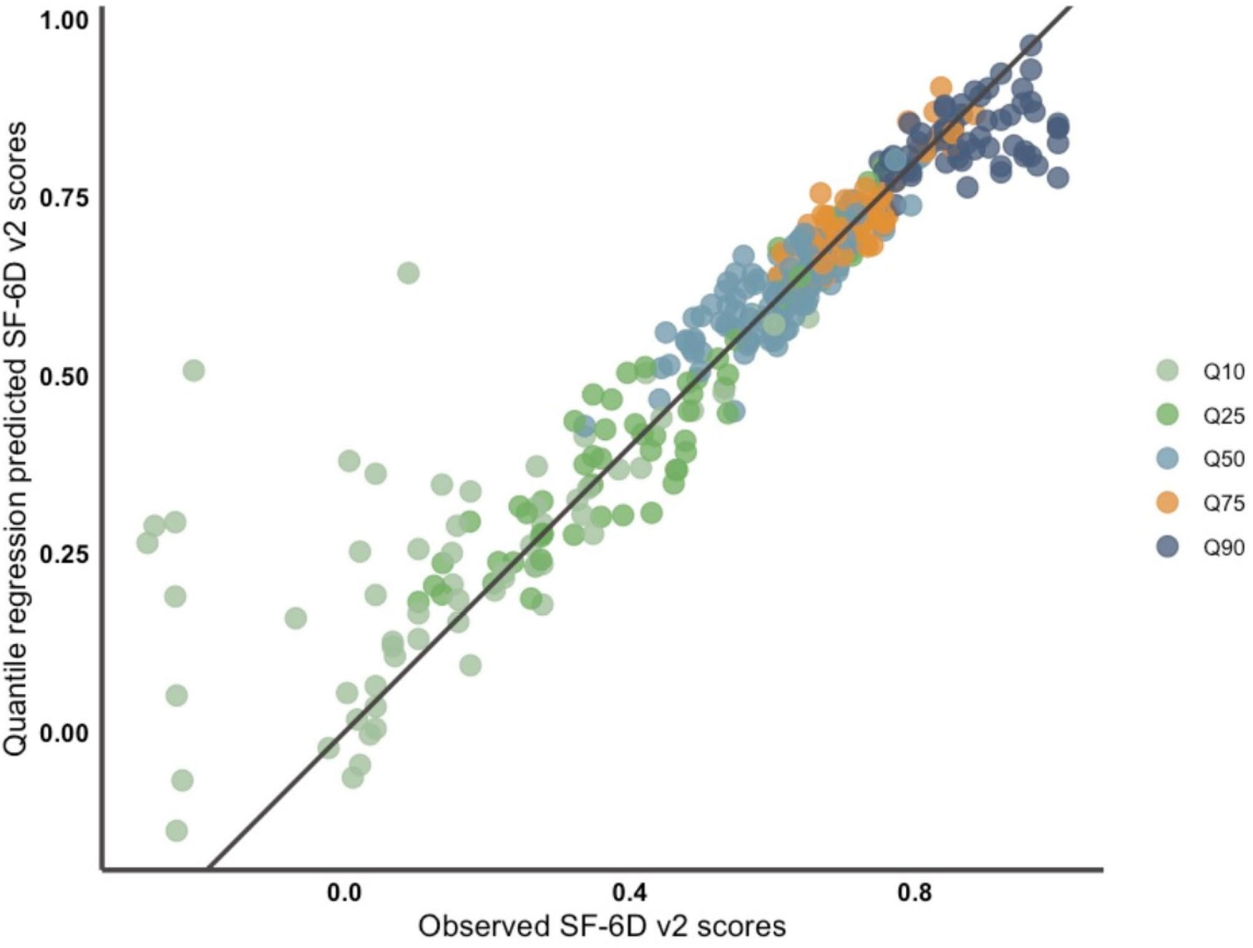
Scatter plot of prediction vs. observation resulting from the quantile regression. **Note. **The figure represents the “best estimations” resulting from the five QR models performed. The legend allows identification of which of the five models provided the “best estimation”. For example, the orange points correspond to estimated values from the QR model with the inference based on the third quartile (Q75)

The SF-6Dv2 scores predicted by the OLS regression varied from 0.3404 to 0.8341, whereas the observed values fell between − 0.277 and 1. The mean and standard deviation of the predicted SF-6Dv2 scores by the OLS model were 0.5683 and 0.0931 (much lower than that of the original distribution, which is 0.2628), respectively. This confirmed that the limited capacity of the OLS regression to estimate lower and higher SF-6Dv2 scores. As a comparison, the SF-6Dv2 scores predicted by the quantile regression model (“best estimate”) varied from − 0.1704 to 0.9781. The “best estimate” from the quantile regression was closer to the reference line y = x than the estimate values derived from the OLS regression, suggesting that the estimate values derived from the quantile regression had a higher accuracy when the information from the five models was fully considered (Figs. 2 and 3).

## Discussion

Our study identified characteristics that were associated with SF-6Dv2 scores in dialysis people in China. The use of quantile regression refined the finding of the OLS regression and increased the understanding of the association between the SF-6Dv2 score and the associated variables. This additional information provided us with models that had more accurate predictive performance compared to the OLS model, which suggests that quantile regression better describes the relationships between the SF-6Dv2 scores and the associated factors.

However, both the OLS and quantile regression models showed poor fit at the lower end of the SF-6Dv2 utility distribution. This may be partially attributed to the distributional characteristics of the data. In each dimension of SF-6Dv2, the proportion of participants reporting the most severe level was consistently the lowest (Table A1), suggesting a limited representation of extremely poor health states in our sample. Such skewed distributions reduce the model’s exposure to low-utility cases during training, thereby impairing its predictive performance in this region.

Sex did not show an association with the SF-6Dv2 scores in our study. Although the association between sex and HRQoL varies among studies, our result was consistent with those of Merkus et al. [22] We found a statistically significant association between SF-6Dv2 scores and age, which was consistent with the results of previous research [22, 23]. Moreover, our research further found that the negative association between age and HRQoL is stronger among patients with relatively low scores, which suggests that dialysis patients with already poor health may be more susceptible to additional health deterioration due to aging. The education level was found to have a statistically significant positive association with the SF-6Dv2 scores in our study, especially when HRQoL is relatively low, which is supported by other studies that suggest that low education level decreases HRQoL in patients with CKD and ESRD [9, 24, 25]. One possible reason for this association is that low education level enhances patients’ anxiety about their illness. At the same time, this study found that the extent of the effect of education level was heterogeneous across HRQoL levels, which helps to explain why Okoro et al. found that education level and quality of life were inversely associated for CKD patients with relatively high HRQoL (0.82 ± 0.13) [13]. 

The effects of economic status related variables and HRQoL were also explored. Currently working and having a monthly income > 8000 RMB were positively associated with the SF-6Dv2 scores, especially when the SF-6Dv2 scores were near the two ends of the distribution. Previous studies have supported the positive association between income level and HRQoL [26–28]. Naimi et al. implied that the positive association between having a job and HRQoL could be due to the healthy worker effect [29], but our results indicated that currently working had stronger association among patients with lower SF-6Dv2 scores, which cannot be fully explained by healthy worker effect. Having a loan due to illness was negatively associated with the SF-6Dv2 scores in our study, especially when the SF-6D v2 scores were relatively low. To our knowledge, the potential relationship between loans and HRQOL has not been previously analyzed in renal disease context. Nevertheless, a US study found that negative financial events such as loans were significantly associated with poorer HRQoL among multiple endocrine neoplasia type 1 patients [30]. This suggests that financial burdens may have a similar detrimental impact on HRQoL in dialysis patients, potentially through increased stress, anxiety, and reduced access to care.

There are several limitations worth noticing. First, part of the variance in the SF-6D scores remained unexplained, showing that there might be some variables other than those controlled being associated with the HRQoL, such as severity of comorbidities. Second, the sample size of the present study was small, which might contribute to the overfitting of the model. We minimized this problem by narrowing the set of predictors with univariate analysis and assessing model performance using cross-validation. Apart from the cross-validation, other validation techniques and algorithms, such as bootstrap methods or Bayesian approaches, could provide additional insights. Third, traditional metrics such as MAE and RMSE mainly capture average prediction errors and may fail to reflect distributional aspects like heteroscedasticity, skewness, or differences in prediction uncertainty across the range of outcomes. Future research could incorporate alternative evaluation metrics.

The implications of this study can be considered from two perspectives. First, from the perspective of decision support, we found that among dialysis patients, negative financial events (such as loans and losing job) were negatively associated with lower SF-6Dv2 scores. This finding supports providing financial assistance and reemployment opportunities to patients with severe conditions. Additionally, low education levels may exacerbate anxiety in dialysis patients with severe conditions, further diminishing their quality of life. Public institutions such as hospitals and governments could consider designing or enhancing educational programs tailored to middle aged and elder patients to improve their understanding of kidney disease and dialysis treatment, or choose to promote renal supportive therapy to patients with poorer health states [31]. Second, from a methodological perspective, this study highlights the advantages of quantile regression over the more commonly used OLS models for modelling HRQoL data. The accuracy of OLS models is limited because the distribution of patients’ HRQoL data may violate the normality assumption required by OLS, and the strength of associations between variables and HRQoL may vary across different quantiles. The results of Walt test in this study provided strong evidence of the heterogeneity of the independent variable’s impact on patients’ quality of life when they were in different positions of the SF-6Dv2 score distribution. As far as we know, this study is the first to use cross-validation to examine the predictive performance of quantile regression compared to OLS in the context of HRQoL, demonstrating that quantile regression can capture more generalizable information about the distribution of HRQoL than OLS. This methodological choice aligns with the clinimetric approach typically adopted in HRQoL measurement, which focuses on developing and validating patient-reported outcome measures that can sensitively and meaningfully reflect subjective health experiences [32, 33]. Such an approach is particularly essential in CKD populations, where even subtle shifts in HRQoL may signal important changes in clinical status and guide more tailored patient care.

## Conclusion

This study highlighted that the size of the associations between potential patient factors and SF-6Dv2 scores varied depending on the point of the quantile distribution considered in Chinese dialysis patients. Future studies could consider using larger sample sizes to confirm the potential associations with different dimensions of HRQoL or using longitudinal datasets to examine the causal effects of different factors on HRQoL.

## Electronic supplementary material

Below is the link to the electronic supplementary material.

Supplementary Material 1

## Data Availability

The datasets generated and/or analysed during the current study are not publicly available due to privacy concerns and confidentiality agreements but are available from the corresponding author on reasonable request.
