# Supplementary material for: Socio-demographic characteristics associated with SF-6D v2 utility scores in patients undergoing dialysis in China: contributions of the quantile regression
Source: Health Qual Life Outcomes. 2025 Jul 28;23:76. doi: 10.1186/s12955-025-02401-y (PMC12305961; doi:10.1186/s12955-025-02401-y)
Supplement: Supplementary file 1 — Supplementary Material 1 [file 12955_2025_2401_MOESM1_ESM.docx]

**Additional File 1**

**Socio-demographic characteristics associated with SF-6D v2 utility scores in middle aged and elder patients undergoing dialysis in China: contributions of the quantile regression**

Zeyuan Chen^1^**,** Li Yang^2^, Ye Zhang^3,4*^

^1^Department of Informatics and Media, Uppsala University, SE-751 05 Uppsala, Sweden

^2^School of Public Health, Peking University, Beijing 100191, People’s Republic of China

^3^Population Development Studies Center, Renmin University of China, Beijing 100872, People’s Republic of China

^4^School of Population and Health, Renmin University of China, Beijing 100872, People’s Republic of China

**Table A1 Distribution across levels of the SF-6Dv2 dimensions**

| SF-6Dv2 | |
| --- | --- |
| Dimensions | Amount |
| Physical functioning |  |
| Level 1 | 121(32%) |
| Level 2 | 112(29.6%) |
| Level 3 | 71(18.8%) |
| Level 4 | 37(9.8%) |
| Level 5 | 37(9.8%) |
| Role limitation |  |
| Level 1 | 131(34.7%) |
| Level 2 | 95(25.1%) |
| Level 3 | 68(18.0%) |
| Level 4 | 42(11.1%) |
| Level 5 | 42(11.1%) |
| Social functioning |  |
| Level 1 | 110(29.1%) |
| Level 2 | 89(23.5%) |
| Level 3 | 83(22.0%) |
| Level 4 | 51(13.5%) |
| Level 5 | 45(11.9%) |
| Pain |  |
| Level 1 | 113(29.9%) |
| Level 2 | 106(28%) |
| Level 3 | 71(18.8%) |
| Level 4 | 59(15.6%) |
| Level 5 | 24(6.3%) |
| Level 6 | 5(1.3%) |
| Mental health |  |
| Level 1 | 123(32.5%) |
| Level 2 | 113(29.9%) |
| Level 3 | 68(18.0%) |
| Level 4 | 57(15.1%) |
| Level 5 | 17(4.5%) |
| Vitality |  |
| Level 1 | 147(38.9%) |
| Level 2 | 96(25.4%) |
| Level 3 | 84(22.2%) |
| Level 4 | 30(7.9%) |
| Level 5 | 21(5.6%) |

**Table A2 Coefficient Estimates (CE) of Quantile Regression Models**

| **Variables** | **CE** | **Upper 95% CI** | **Lower 95% CI** | | **P value** |
| --- | --- | --- | --- | --- | --- |
| **Q10** | | | | | |
| Constant | 0.3425 | 0.2571 | 0.5049 | | **0.0166** |
| Education level | 0.0601 | 0.0106 | 0.0959 | | 0.1111 |
| Monthly income > 8000 | 0.0466 | 0.0191 | 0.1846 | | 0.6118 |
| Age | -0.0035 | -0.0048 | -0.0020 | **0.0552** | |
| Currently working | 0.1634 | -0.0039 | 0.2485 | **0.0773** | |
| Number of comorbidities | -0.0679 | -0.1042 | -0.0217 | **0.0208** | |
| Having a loan due to illness | -0.0745 | -0.1712 | -0.0113 | 0.2212 | |
| **Q25** | | | | | |
| Constant | 0.7816 | 0.4518 | 0.9121 | **0.0138** | |
| Education level | 0.0090 | -0.0336 | 0.0732 | **0.0925** | |
| Monthly income > 8000 | 0.0329 | -0.0519 | 0.1209 | 0.6171 | |
| Age | -0.0057 | -0.0070 | -0.0011 | **0.0326** | |
| Currently working | 0.1232 | 0.0079 | 0.2052 | **0.0837** | |
| Number of comorbidities | -0.0534 | -0.1048 | -0.0288 | **0.0152** | |
| Having a loan due to illness | -0.0918 | -0.1830 | -0.0098 | 0.2022 | |
| **Q50** | | | | | |
| Constant | 0.7219 | 0.6377 | 0.8609 | **0.0068** | |
| Education level | 0.0185 | -0.0089 | 0.0382 | **0.0927** | |
| Monthly income > 8000 | -0.0032 | -0.0579 | 0.0295 | 0.5269 | |
| Age | -0.0016 | -0.0038 | -0.0003 | **0.0171** | |
| Currently working | 0.0742 | 0.0324 | 0.1088 | 0.1270 | |
| Number of comorbidities | -0.0468 | -0.0625 | -0.0230 | **0.0177** | |
| Having a loan due to illness | -0.0321 | -0.0680 | 0.0081 | 0.2250 | |
| **Q75** | | | | | |
| Constant | 0.8296 | 0.7351 | 0.9810 | **0.0173** | |
| Education level | -0.0014 | -0.0178 | 0.0150 | 0.1069 | |
| Monthly income > 8000 | 0.0389 | -0.0479 | 0.0845 | 0.5804 | |
| Age | -0.0017 | -0.0036 | -0.0004 | **0.0284** | |
| Currently working | 0.1226 | 0.0468 | 0.1596 | **0.0808** | |
| Number of comorbidities | -0.0228 | -0.0484 | -0.0046 | **0.0143** | |
| Having a loan due to illness | -0.0026 | -0.0596 | 0.0275 | 0.2017 | |
| **Q90** | | | | | |
| Constant | 0.8656 | 0.7603 | 1.1951 | **0.0025** | |
| Education level | 0.0114 | -0.0314 | 0.0385 | **0.0576** | |
| Monthly income > 8000 | 0.0455 | -0.0383 | 0.1163 | 0.5699 | |
| Age | -0.0012 | -0.0048 | 0.0014 | **0.0190** | |
| Currently working | 0.0611 | 0.0245 | 0.1386 | 0.1192 | |
| Number of comorbidities | -0.0200 | -0.0524 | 0.0033 | **0.0157** | |
| Having a loan due to illness | 0.0123 | -0.0825 | 0.0618 | 0.1575 | |

Note: *CE* Coefficient Estimates

**Table A3 Results of Wald Test**

| **Variable** | **F value** | **Pr(>F)** |
| --- | --- | --- |
| **Q10 and Q25** | | |
| Education level | 4.02952 | 0.04507 * |
| Monthly income > 8000 | 0.08009 | 0.77726 |
| Age | 1.49541 | 0.22176 |
| Currently working | 0.34523 | 0.55700 |
| Number of comorbidities | 0.41611 | 0.51908 |
| Having a loan due to illness | 0.08531 | 0.77030 |
| **Q10 and Q50** | | |
| Education level | 3.16620 | 0.07558 . |
| Monthly income > 8000 | 1.38850 | 0.23903 |
| Age | 1.46423 | 0.22664 |
| Currently working | 1.38521 | 0.23959 |
| Number of comorbidities | 1.03895 | 0.30839 |
| Having a loan due to illness | 0.42661 | 0.51385 |
| **Q10 and Q75** | | |
| Education level | 6.20963 | 0.01292 * |
| Monthly income > 8000 | 0.01858 | 0.89162 |
| Age | 1.26021 | 0.26197 |
| Currently working | 0.25392 | 0.61448 |
| Number of comorbidities | 4.35767 | 0.03718 |
| Having a loan due to illness | 1.11215 | 0.29195 |
| **Q10 and Q90** | | |
| Education level | 2.38235 | 0.12313 |
| Monthly income > 8000 | 0.00036 | 0.98489 |
| Age | 1.09102 | 0.29658 |
| Currently working | 1.45265 | 0.22848 |
| Number of comorbidities | 3.20874 | 0.07365 * |
| Having a loan due to illness | 1.29875 | 0.25480 |
| **Q25 and Q50** | | |
| Education level | 0.14882 | 0.69978 |
| Monthly income > 8000 | 0.57583 | 0.44819 |
| Age | 5.34292 | 0.02107 * |
| Currently working | 1.26532 | 0.26100 |
| Number of comorbidities | 0.09987 | 0.75207 |
| Having a loan due to illness | 1.79657 | 0.18053 |
| **Q25 and Q75** | | |
| Education level | 0.13508 | 0.71333 |
| Monthly income > 8000 | 0.00991 | 0.92071 |
| Age | 3.96533 | 0.04681 * |
| Currently working | 0.00010 | 0.99197 |
| Number of comorbidities | 1.59199 | 0.20743 |
| Having a loan due to illness | 3.00356 | 0.08349 . |
| **Q25 and Q90** | | |
| Education level | 0.00505 | 0.94335 |
| Monthly income > 8000 | 0.03593 | 0.84971 |
| Age | 3.35307 | 0.06747 . |
| Currently working | 1.10889 | 0.29266 |
| Number of comorbidities | 1.34258 | 0.24695 |
| Having a loan due to illness | 2.84982 | 0.09180 . |
| **Q50 and Q75** | | |
| Education level | 3.32085 | 0.06880 |
| Monthly income > 8000 | 1.19003 | 0.27567 |
| Age | 0.00555 | 0.94064 |
| Currently working | 2.62041 | 0.10591 |
| Number of comorbidities | 5.08803 | 0.02438 * |
| Having a loan due to illness | 1.36845 | 0.24245 |
| **Q50 and Q90** | | |
| Education level | 0.10280 | 0.74859 |
| Monthly income > 8000 | 1.13900 | 0.28620 |
| Age | 0.05130 | 0.82087 |
| Currently working | 0.11649 | 0.73297 |
| Number of comorbidities | 1.98477 | 0.15930 |
| Having a loan due to illness | 1.10412 | 0.29370 |
| **Q75 and Q90** | | |
| Education level | 0.44721 | 0.50387 |
| Monthly income > 8000 | 0.02346 | 0.87831 |
| Age | 0.09334 | 0.76006 |
| Currently working | 3.12796 | 0.07736 . |
| Number of comorbidities | 0.03374 | 0.85432 |
| Having a loan due to illness | 0.19055 | 0.66258 |

Note: . p value < 0.1; * p value < 0.05; ** p value < 0.01
